# Gaseous nitric oxide tumor ablation induces an anti-tumor abscopal effect

**DOI:** 10.1186/s12935-022-02828-z

**Published:** 2022-12-13

**Authors:** Hila Confino, Frederick M. Dirbas, Matan Goldshtein, Shay Yarkoni, Rinat Kalaora, Meital Hatan, Shani Puyesky, Yakir Levi, Lidor Malka, Matt Johnson, Selena Chaisson, Jedidiah M. Monson, Amir Avniel, Steve Lisi, David Greenberg, Ido Wolf

**Affiliations:** 1 Beyond Cancer Ltd., 7608801 Rehovot, Israel; 2 Department of General Surgery, Stanford University, Stanford, CA USA; 3 Beyond Air Ltd., 7608801 Rehovot, Israel; 4 Beyond Cancer Ltd., Atlanta, GA USA; 5 California Cancer Associates for Research and Excellence, Fresno, CA USA; 6 Beyond Air Inc, Garden City, NY 11530 USA; 7 Oncology Division, Tel Aviv Sourasky Medical Center, Tel Aviv, Israel; 8 Sackler School of Medicine, Tel Aviv University, Tel Aviv, Israel

**Keywords:** Gaseous nitric oxide, Cancer, Metastasis, Immunotherapy, Solid tumors

## Abstract

**Background:**

In-situ tumor ablation provides the immune system with the appropriate antigens to induce anti-tumor immunity. Here, we present an innovative technique for generating anti-tumor immunity by delivering exogenous ultra-high concentration (> 10,000 ppm) gaseous nitric oxide (UHCgNO) intratumorally.

**Methods:**

The capability of UHCgNO to induce apoptosis was tested in vitro in mouse colon (CT26), breast (4T1) and Lewis lung carcinoma (LLC-1) cancer cell lines. In vivo*,* UHCgNO was studied by treating CT26 tumor-bearing mice in-situ and assessing the immune response using a Challenge assay.

**Results:**

Exposing CT26, 4T1 and LLC-1 cell lines to UHCgNO for 10 s–2.5 min induced cellular apoptosis 24 h after exposure. Treating CT26 tumors in-situ with UHCgNO followed by surgical resection 14 days later resulted in a significant secondary anti-tumor effect in vivo. 100% of tumor-bearing mice treated with 50,000 ppm UHCgNO and 64% of mice treated with 20,000 ppm UHCgNO rejected a second tumor inoculation, compared to 0% in the naive control for 70 days. Additionally, more dendrocytes infiltrated the tumor 14 days post UHCgNO treatment versus the nitrogen control. Moreover, T-cell penetration into the primary tumor was observed in a dose-dependent manner. Systemic increases in T- and B-cells were seen in UHCgNO-treated mice compared to nitrogen control. Furthermore, polymorphonuclear-myeloid-derived suppressor cells were downregulated in the spleen in the UHCgNO-treated groups.

**Conclusions:**

Taken together, our data demonstrate that UHCgNO followed by the surgical removal of the primary tumor 14 days later induces a strong and potent anti-tumor response.

## Introduction

Metastases are responsible for the majority of cancer-related deaths [1]. Therefore, a primary goal of cancer therapy is to prevent metastases formation and eradicate overt metastases [2, 3]. When only a small number of metastases are present, a condition known as oligometastatic disease, these metastases can be treated locally by surgery, radiation therapy, or local therapy. Yet, in most circumstances, patients harbor a large number of metastases, many of which are too small to be detected by the current imaging modalities, thus requiring systemic treatment. However, the efficacy of systemic treatment of most metastatic solid tumors is limited, and although they can prolong life to some extent and improve quality of life, they are not curative.

In recent years, immune checkpoint inhibitors (ICI) revolutionized the treatment of several solid tumor types, most notably melanoma and microsatellite instability (MSI) high tumors [4]. However, in most other indications, including non-small cell lung cancer (NSCLC), ICI only prolongs survival to a limited extent [5]. Accumulating data suggest that local tumor ablation may increase sensitivity to ICI, even in resistant tumors [6]. Tumor ablation destroys residual malignant cells in primary tumors and distant metastases, resulting in the accumulation of cancerous debris in-situ*,* including tumor antigens and damage-associated molecular patterns (DAMPs) [6, 7]. Antigen-presenting cells, activated by tumor antigens and DAMPs, can then present these molecules to immune effector cells, thereby triggering an adaptive anti-tumor immune response [8–10]. Local tumor ablation can be achieved using a plethora of minimally invasive techniques, including Stereotactic body radiation therapy (SBRT), radiofrequency, cryoablation, focused ultrasound, and microwave ablation [11, 12]. These techniques are commonly used to treat early-stage hepatocellular carcinoma (HCC), as well as liver and lung metastases of various primary tumors [13]. Importantly, these ablation techniques trigger an immune response [8, 11].

Recently, ultra-high concentration gaseous nitric oxide (UHCgNO) has emerged as a novel method for tumor ablation. While low concentrations of NO gas (gNO) may possess pro-oncogenic properties, preclinical studies using high concentrations have shown its potential as a potent anti-neoplastic agent [14–19]. This anti-neoplastic effect may be mediated by reactive NO species generation, such as peroxynitrite, that can oxidize DNA and induce single-strand breaks [16]. In addition, UHCgNO causes cell death via necrosis and apoptosis. NO-mediated apoptosis involves the accumulation of the tumor suppressor protein p53, mitochondrial damage, alterations in the expression of members of the anti-apoptotic Bcl-2 family, caspase activation, and DNA fragmentation [15, 16]. Therefore, endogenous NO generation in tumor-adjacent blood vessels may limit cancer cells’ intravasation by causing DNA damage and apoptosis of malignant cells [16]. Importantly, gNO can also sensitize resistant cancer cells to chemotherapy and radiotherapy by improving tumor blood flow and, therefore, drug and oxygen delivery [19] and by increasing DNA double-strand breaks [20]. Thus, high dose NO can potentially serve as an anti-invasion, anti-metastatic, and anti-angiogenic agent [16, 20]. Investigational therapies based on NO-releasing molecules [21] are limited by the low levels of NO attained by these proposed modalities versus the direct exposure of the tumor to UHCgNO.

UHCgNO-based ablation has several advantages over other modes of ablation. The highly diffusible nature of gNO may increase its distribution within the tumor, while its short half-life will limit its effect to the tumor [22] and short-term treatment is sufficient to stimulate an immune-mediated anti-tumor response.

Currently, low NO concentrations are approved as an inhaled drug for treating term and near-term (> 34 weeks gestation) neonates with hypoxic respiratory failure (HRF) associated with pulmonary hypertension [23]. Additionally, in Europe, NO is administered prior to cardiac surgery to improve pulmonary artery pressure and outcomes [24]. Additionally, NO is used off-label as a potent bactericidal, fungicidal, and virucidal activity for the treatment of *Pseudomonas aeruginosa*, Methicillin-resistant *Staphylococcus aureus* (MRSA), *Candida albicans* [25, 26], and Novel Coronavirus [27].

Here we present a novel treatment paradigm involving in-situ tumor ablation with UHCgNO. Treating tumors in-situ with UHCgNO showed significant secondary anti-tumor effects in vivo, with mice rejecting a second tumor inoculation. Additionally, UHCgNO treatment resulted in an enhanced immune profile within the tumor microenvironment and systemically. Taken together, our data demonstrate the potential of UHCgNO as a treatment for systemic metastases via induction of an anti-tumor immune response and uncovers part of the mechanism underlying UHCgNO tumor ablation.

## Methods

### NO gas

10,000–100,000 ppm NO was administered from 3 L cylinders, with Nitrogen serving as its stabilizing gas (Gordon Gas and Chemical, Tel Aviv, Israel). Nitrogen supplied from 3 L cylinders served as control gases. All procedures were performed in a chemical hood. Gases were delivered via a pressure regulator through a PVC hose (International Biomedical, USA). The flow rate was set to 0.2 (for in vivo studies) -1.0 (for in vitro studies) liters per minute (LPM) using a manual flow meter.

### Tumor cell lines

Mouse CT26.WT colon, 4T1 breast and LLC-1 Lewis lung carcinoma cancer cell lines were purchased from an American Type Culture Collection (ATCC) local distributor, Sartorius (Beit Haemek, Israel). CT26 and 4T1 cell lines were grown in RPMI-based media (ATCC) supplemented with 10% fetal bovine serum and 1% penicillin–streptomycin (Sartorius). LLC-1 cells were grown in DMEM medium (ATCC) supplemented with 10% fetal bovine serum and 1% penicillin–streptomycin (Sartorius).

### Preparation of tumor cells

Tumor cell suspensions were prepared in a cell culture medium or Hanks’ Balanced Salt Solution (HBSS, Sartorius) at concentrations of 1 × 10^5^ cells/ml for in vitro studies or 5.0–10.0 × 10^6^ cells/ml for in vivo studies. Freshly prepared cells were grown to 70% confluency, harvested using trypsin (Sartorius), and counted using a hemocytometer.

### In vitro studies

Cell viability was assessed following direct exposure of cancer cells to gNO in 96-well plates. Exposure of gNO at 10,000–100,000 ppm or air was delivered at 1.0 LPM in a 1.7 L box. For gNO treatment, cell culture media was removed, and cells were exposed to gNO for up to three minutes. Immediately following gas exposure, cell culture medium was added, and cells were incubated at 37 °C in 5% CO_2_ overnight. Cell viability was assessed using XTT (Sartorius) and Annexin V–Propidium Iodide (Miltenyi-Biotech) assays.

### In vivo experiments

Study overview: CT26 cells were inoculated subcutaneously (s.c.) on the right flank of 8–10 weeks old male Balb/c mice (Envigo, Israel, and the Netherlands) at a concentration of 5.0 × 10^5^ CT26 cells in 100 µL HBSS. Treatment was initiated once tumors reached an average volume of ~ 50 mm^3^ (around 10 days following tumor inoculation). Mice were evaluated for tumor volume using a digital caliper up to 14 days post-gNO treatment, and tumors were surgically removed. Seven days later (21 days post-gNO treatment of primary tumors), mice were inoculated with challenge tumor cells on the contralateral (left) flank. Primary tumor recurrence rate and challenge tumor take were evaluated up to the excision day and 75 days post-challenge inoculation, respectively.

### In vivo gNO treatment

Before each treatment, mice were anesthetized by an intraperitoneal (i.p.) injection of 100 mg/kg ketamine (Zoetis) and 10–20 mg/kg xylazine hydrochloride solution (Abic). After 10 min, mice were treated by means of intratumoral delivery of 20,000 or 50,000 ppm gNO. The needle was inserted into the tumor horizontally such that it was located approximately in the tumor’s center (about half of the tumor diameter, depending on tumor size and shape). Gas was injected for 5 min using a 23G needle. The outlet pressure was set to ~ 3.5 bar using a pressure regulator connected to the cylinder. A stainless-steel PTFE coated hose was connected to the pressure regulator on one end and a manual flow meter at the other end. A PVC hose was connected to the flow controller and a 23G hypodermic needle. The treatment regimen was 20,000–50,000 ppm NO at 0.2LPM rate for 5 min using a manual flow meter. All primary tumors were resected 14 days post-gNO treatment. A week after tumor resection, all mice were re-challenged with a second dose of CT26 cells.

### In vivo nitrogen treatment

Nitrogen is the balance gas for NO and was used as a control treatment. Before each treatment, mice were anesthetized by an intraperitoneal (i.p.) injection of 100 mg/kg ketamine (Zoetis) and 10–20 mg/kg xylazine hydrochloride solution (Abic). After 10 min, mice were treated with intratumor nitrogen. The needle was inserted into the tumor horizontally to place it in the tumor’s center (about half of the tumor diameter, depending on tumor size and shape). Gas was injected for 5 min using a 23G needle. The outlet pressure was set to ~ 3.5 bar using a pressure regulator connected to the cylinder. A stainless-steel PTFE-coated hose was connected to the pressure regulator on one end and a manual flow meter on the other end. A PVC hose was connected to the flow controller and a 23G hypodermic needle. The nitrogen treatment was delivered at 0.2LPM rate for 5 min using a manual flow meter. All primary tumors were resected 14 days post-nitrogen treatment. A week after tumor resection, all mice were re-challenged with a second dose of CT26 cells.

### Tumor volume calculation

Local tumor growth was determined by measuring 3 mutually orthogonal tumor dimensions 2–3 times per week, according to the following formula:

$${\text{Tumor Volume }} = \frac{\pi }{6} \times \left[ {{\text{Diameter 1}} \times {\text{ Diameter 2}} \times {\text{ Diameter 3}}} \right]$$

### Primary tumor excision

Fourteen days after NO treatment of CT26 tumor-bearing mice, all remaining tumors were excised. The tumor of one mouse in the 20,000 ppm NO group was excised 10 days post-treatment due to tumor size. This mouse was excluded from the average primary CT26 tumor volume graph but included in challenge assay graphs. Briefly, all surgery tools (Vet Market, Israel) were washed with Septal Scrub (Teva, Israel) and heat disinfected, followed by Ethanol 70% (Romical, Israel) disinfection. Mice were anesthetized by an i.p injection of 100 mg/kg ketamine and 20 mg/kg xylazine hydrochloride solution. Local anesthesia using 0.5% Lidocaine cream (Vet Market, Israel) was applied to the tumor surface. After 10 min, the tumor was disinfected by gauze pads (Life, Israel) saturated with Ethanol 70%. The tumor was stabilized using a sterile tweezer and detached from the skin using sterile scissors. The operated tumor site was disinfected via local administration of Polydine (Teva, Israel) and Ethanol 70% (Romical, Israel). The skin was fused using medical clips (Bar Naor, Israel). After treatment, all mice were closely monitored until complete recovery. Dipyrone (Teva, Israel) was administered in drinking water for 3 days (0.4 g/200 ml drinking water).

### Challenge tumor inoculation

On day 21 post-gNO treatment of the primary tumor, the appropriate cancer cell suspensions were prepared, and s.c. cell inoculation was repeated on the contralateral (left) flank. The appearance of a second induced tumor (challenge tumor) was monitored 2–3 times a week by visual and palpable observation. Naïve or nitrogen-treated mice inoculated with tumor cells served as controls.

### Conditions for terminating the participation of a particular animal in the experiment

Animals found in a moribund condition or showing severe pain and enduring signs of severe distress were humanely euthanized. The health condition of the animals was assessed via the below mouse distress scoring:

1. Appearance: normal—0, coat staring, ocular or nasal discharge-1, piloerection—2, hunched up—3.
2. Hydration status: normal—0, skin tents when pinched quickly recovers—1, skin tents when pinched slowly recovers—2, skin remains tented severe dehydration -3.
3. Natural Behavior: normal, i.e., active—0, less mobile and alert—1, isolated—2, restless/shivering/very still—3
4. Body weight: not different from controls—0, weight loss of 0–10%—1, weight loss of 10–20%, weight loss over 20% -3.
5. Tumor volume (of all tumors): 500–1,000 mm^3^—1; 1,000–1,500 mm^3^—2; > 1,500 mm^3^—animals with a > 1,500 mm^3^ tumor were humanely euthanized immediately.

When the total score is  ≥ 7, the mouse was assessed 1–2 times a day, and wet food was placed at the bottom of the cage. When the total score was 10 or the total tumor burden exceeded 1,500 mm^3^, the mouse was euthanized by cervical dislocation. Briefly, the mouse was first anesthetized using Ketamine and Xylazine anesthetic mix as previously described, followed by cervical dislocation. When animals were euthanized for humane reasons or found dead, the time of death was recorded as precisely as possible. The conditions for animal sacrifice described above are reflected as the endpoint for the survival experiment.

### Histology and immunohistochemistry

Colon tumor samples from mice were harvested, fixed in 4% formaldehyde, sent to a Contract Research Organization (CRO), and kept in the fixative for 48 h. The tissues were processed for paraffin embedding using one cassette per animal. 4 μm paraffin sections were cut, placed on glass slides, and stained with Hematoxylin & Eosin (H&E) for general histopathology and immunohistochemistry (IHC) using the following antibodies: BD pharmigen, Cat#550286), CD3 (T-lymphocytes, Abcam Cat#Ab16669), CD4 (helper T-lymphocytes, Abcam, Cat#: 183685), CD8 (effector/cytotoxic T-lymphocytes, Novusbio, Cat#NBP2-29475), and CD11b (dendrocytes, Proteintech, Cat#17342).

The inflammation and lymphocyte infiltration levels were histologically assessed by an experienced veterinary pathologist (Pathologica, Israel). Semi-quantitative analysis of pathological changes in H&E-stained sections was performed using a scoring system for the presence and severity of pathological changes in 10 non-overlapping fields, as follows: Immune cells level: 0: no positive cells, 1: < 5 cells positive cells, 2: Very mild staining (5 to  < 15 cells), 3: Mild staining (15 to  < 25 cells), 4: Moderate staining (25 to  < 50 cells), 5: Marked staining (≥ 50 cells). Inflammation was scored as: 0: Scant or absence of inflammatory cells, 1: Inflammatory cells present but markedly less than tumor cells, 2: Inflammatory cell number roughly equal to tumor cells, 3: Predominantly inflammatory cells. Lymphocyte infiltration was scored as: 0: Absence of lymphocytes, 1: < 5 lymphocytes per field, 2: 5 to  < 20 lymphocytes per field, 3: ≥ 20 lymphocytes per field.

Continuous outcomes were analyzed by analysis of variance (ANOVA). The between-group difference was derived from ANOVA with treatment as a factor.

### Flow cytometry analysis

CT26 tumor-bearing mice were treated with 50,000 ppm gNO, 20,000 ppm gNO or nitrogen. Fourteen days post gas treatment all tumors were resected. Mouse spleens were dissociated at day 21 post gas treatment with the GentleMACS Octo (Miltenyi-Biotech). Mouse blood samples were processed 21 days post gas treatment using RBC Lysis Solution (cat#130-094-183, Miltenyi-Biotech). Extracellular labeling of T-cells was performed with FITC anti-CD3 (cat#130-119-758, Miltenyi-Biotech), VioGreen anti-CD4 (cat#130-118-693, Miltenyi-Biotech), and APC-Vio770 anti-CD8 (cat#130–120-806, Miltenyi-Biotech) antibodies. Extracellular labeling of B-cells was performed with PE-Vio615 anti-CD19 (cat#130-111-890, Miltenyi-Biotech) antibodies. Extracellular labeling of polymorphonuclear myeloid-derived suppressor cells (MDSCs) was performed with FITC anti-CD3 (cat#130-119-758, Miltenyi-Biotech) and PE-Vio615 anti-CD19 (cat#130-111-890, Miltenyi-Biotech) for negative staining, VioGreen anti-CD11 (cat#130-113-811, Miltenyi-Biotech), PE Ly-6G (cat#130-123-780, Miltenyi-Biotech) antibodies for positive staining and VioBlue anti-Ly6C (cat#130-111-921, Miltenyi-Biotech) for low staining. Samples were analyzed using MacsQuant^™^ 16 Flow cytometer.

### IFNγ assay

Mouse spleens were dissociated with GentleMACS Octo (Miltenyi-Biotech). T-cells were activated overnight in TexMACS + 10% FBS medium. 2 µL/mL Cell stimulation cocktail (cat#TNB-4970-UL100, Tonbo-Biosciences) was added for 2 h at 37 °C. 1µL/mL Brefeldin A solution (cat# TNB-4506-L001, Tonbo-Biosciences) was then added for 2 h at 37 °C. Extracellular labeling of T-cells was performed with FITC anti-CD3 (cat#130-119-758, Miltenyi-Biotech), VioGreen anti-CD4 (cat#130-118-693, Miltenyi-Biotech), and APC-Vio770 anti-CD8 (cat#130-120-806, Miltenyi-Biotech) antibodies. After fixation and permeabilization of cells, intracellular labeling of T-cells was performed with APC anti-IFNγ antibody (cat#130-123-283, Miltenyi-Biotech). Samples were analyzed using MacsQuant™ 16 Flow cytometer.

### Statistical analysis

Statistical analysis was performed using Excel (Microsoft, USA) or GraphPad Prism 9.3.1 (GraphPad Software, USA) with P < 0.05 considered statistically significant unless stated otherwise.

## Results

### Short exposure to UHCgNO reduces CT26, 4T1, and LLC-1 cell viability

**Fig. 1:**
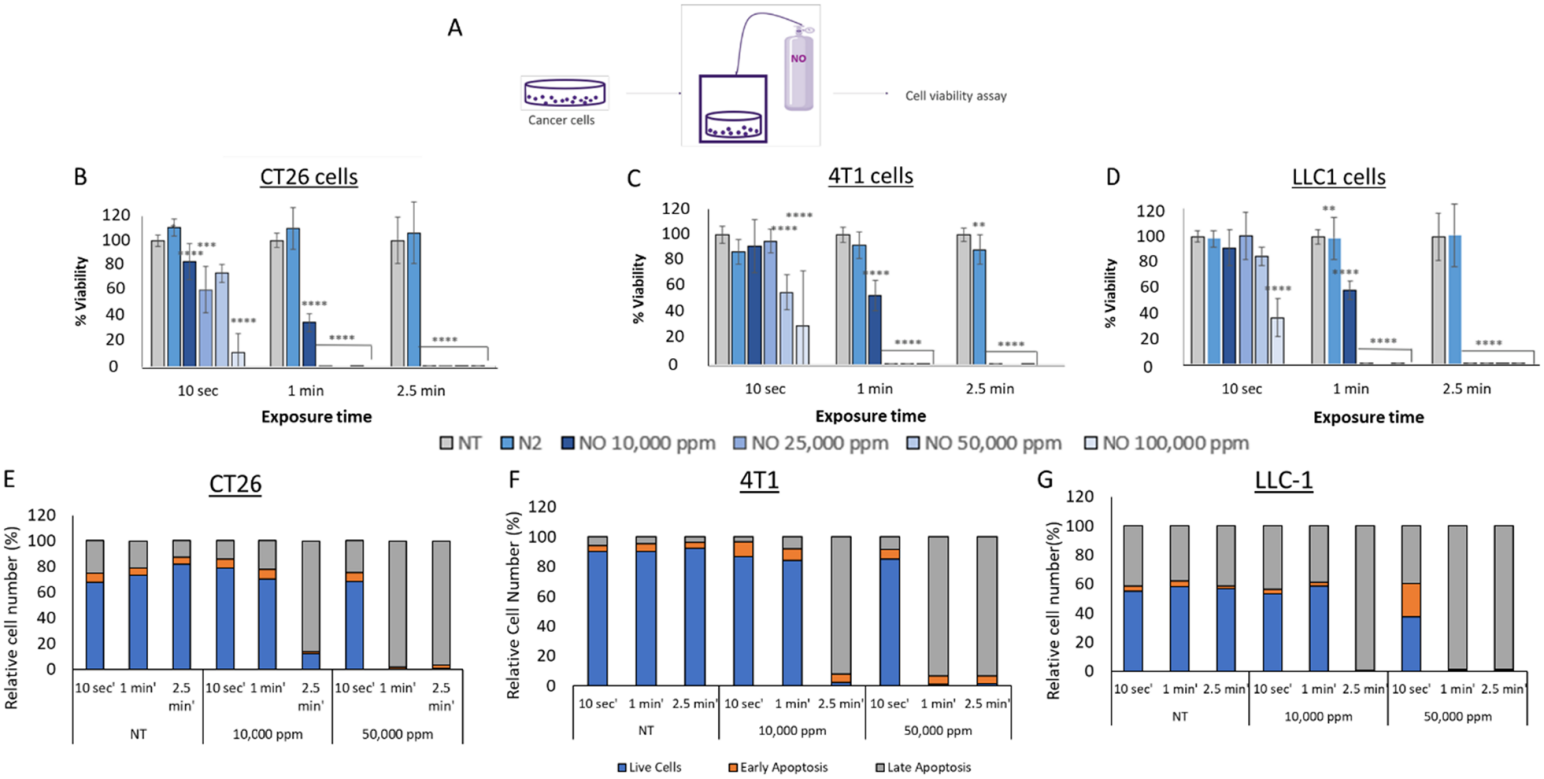
CT26, 4T1 and LLC-1 cell viability after exposure to 10,000–100,000 ppm gNO. **A** Assay scheme **B**–**D** Cancer cells were exposed to 10,000–100,000 ppm gNO or untreated cells for 10 s, 1 min, or 2.5 min. Cancer cell viability was measured after an overnight incubation in XTT proliferation assay. Assay scheme, **B** CT26 cell viability after exposure to 10,000–100,000 ppm gNO. **C** 4T1 cell viability after exposure to 10,000–100,000 ppm gNO. **D** LLC1 cell viability after exposure to 10,000–100,000 ppm gNO. Statistical significance between the groups was determined using One-way ANOVA with Dunnett’s multiple comparison test with Non treated group serving as the control. *P < 0.05, **P < 0.01, ***P < 0.001, ****P < 0.001 **E**–**G** The percentage of cells found at different cell death stages 24 h after a 3 min exposure to air or 50,000 ppm gNO. After ON incubation, CT26, 4T1 and LLC-1 cells were assessed for cell viability. Cell viability was assessed by Annexin V-PI fluorescence analysis 24 h after exposure

We first examined the effects of UHCgNO on cancer cell viability in vitro. CT26, 4T1, and LLC-1 cell lines were exposed to gNO, and proliferation was evaluated using the XTT assay. Exposing CT26, 4T1 and LLC1 cells to 25,000 ppm gNO for one minute significantly reduced their metabolic activity and viability (Fig. 1B–D). The cell populations were next characterized according to cell death markers 24 h after exposure to 10,000 and 50,000 ppm gNO for 10 s, 1, and 2.5 min. Annexin V-PI cell death markers analysis indicated that ~ 88% of CT26 cells, ~ 95% of 4T1 cells, and ~ 99% of LLC-1 cells were apoptotic after 2.5 min exposure to 10,000 ppm gNO while ~ 99% of CT26 cells, ~ 99% of 4T1 cells, and ~ 99% of LLC-1 cells were apoptotic after 2.5-min exposure to 50,000 ppm gNO. When exposed to 10,000 ppm gNO for 2.5 min, 86% of CT26 cells, 93% of 4T1, and 99% of LLC-1 cells expressed late apoptotic markers. When exposure was for 50,000 ppm gNO for 2.5 min the percent of cells expressing late apoptotic markers was 97% of CT26 cells, 93% of 4T1, and 99% of LLC-1 (Fig. 1E-G).

### UHCgNO treatment slows CT26 tumor growth in vivo

**Fig. 2:**
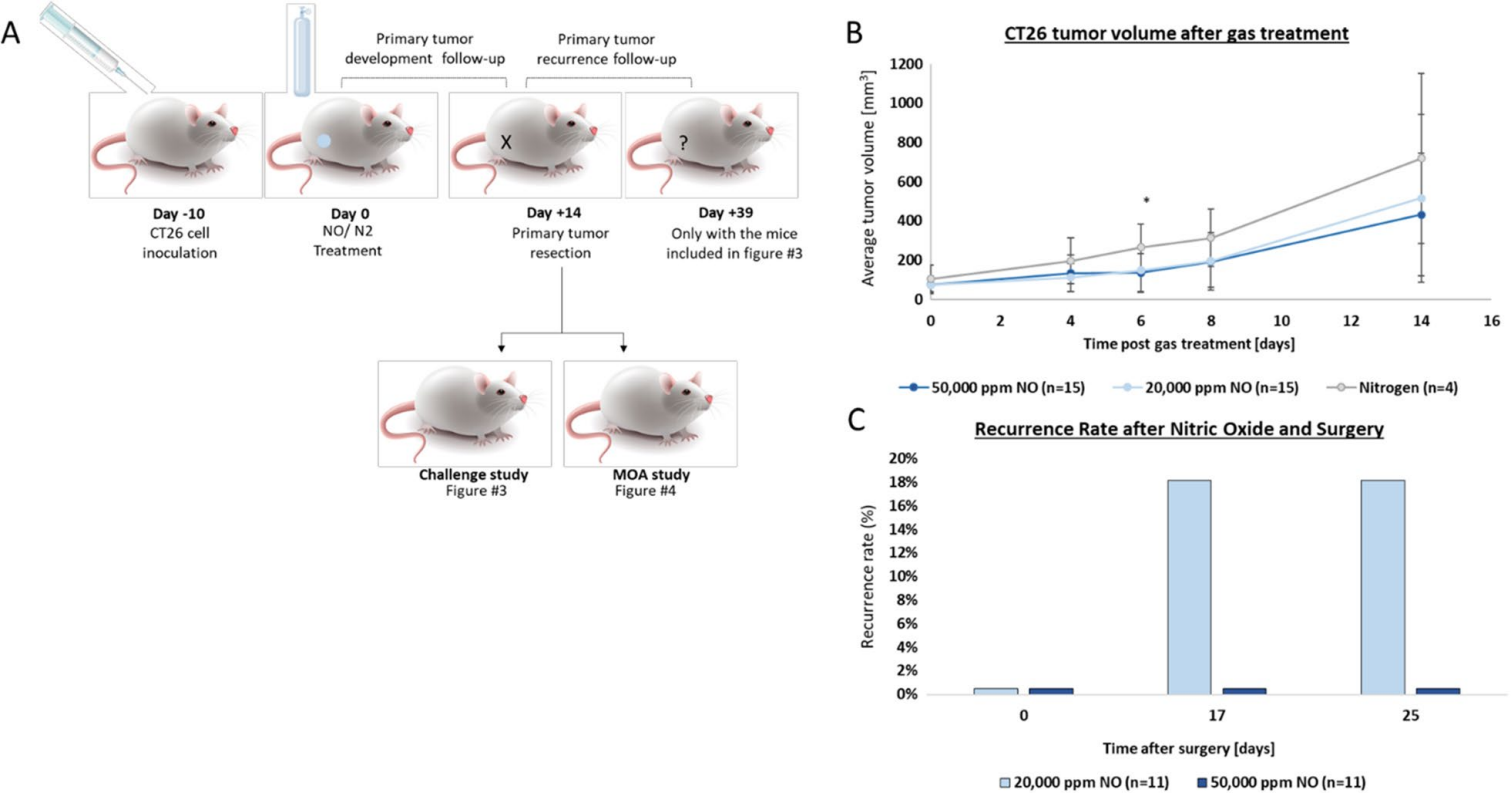
The local and systemic effect of UHCgNO on CT26 tumor-bearing mice. **A** Assay scheme. **B** Tumor growth curves of CT26 tumor-bearing mice (average tumor volume on treatment day 78.61 ± 43.57 mm^3^) treated with 20,000 ppm or 50,000 ppm gNO or nitrogen for 5 min administered intratumorally by a 23G hypodermic needle. Statistical significance between the groups was determined using T-test. 50,000 ppm gNO *versus* nitrogen *P = 0.04; 20,000 ppm gNO *versus* nitrogen *P* = 0.08. **C** The percentage of recurrence after surgery. Fourteen days post-UHCgNO treatment, all primary tumors were excised, and the recurrence rate was monitored. In **C** we have included all mice that had their primary tumors resected at day 14 and had a follow-up time of at least 25 days post primary tumor resection

As CT26 cells showed increased sensitivity in vitro, these cells were selected for the in vivo studies. CT26 cells were injected into the flank of immunocompetent mice. When tumors reached an average size of 50 mm^3^, tumors were intratumorally injected with 20,000 or 50,000 ppm gNO (n = 15 for each group) for 5 min. As controls, mice were treated with nitrogen delivered for 5 min to the tumor. Fourteen days post-treatment, the primary tumor site was examined, and residual cancerous tissue was excised (Fig. 2A). UHCgNO-treated mice at all treatment concentrations presented slower tumor growth compared to those treated with nitrogen. The average tumor volume of mice treated with 50,000 ppm was significantly smaller than that of the control group 14 days post-treatment (Fig. 2B, *P* = 0.04).

We next examined whether treatment with UHCgNO reduced the risk of primary tumor recurrence following tumor excision. Indeed, in mice treated with 50,000 ppm, no recurrence was observed up to 25 days after tumor resection, while in those treated with 20,000 ppm gNO, tumor recurrence was seen in 18% of mice (Fig. 2C, *P* = 0.15).

### Intratumoral treatment reduces mouse susceptibility to tumor challenge

**Fig. 3:**
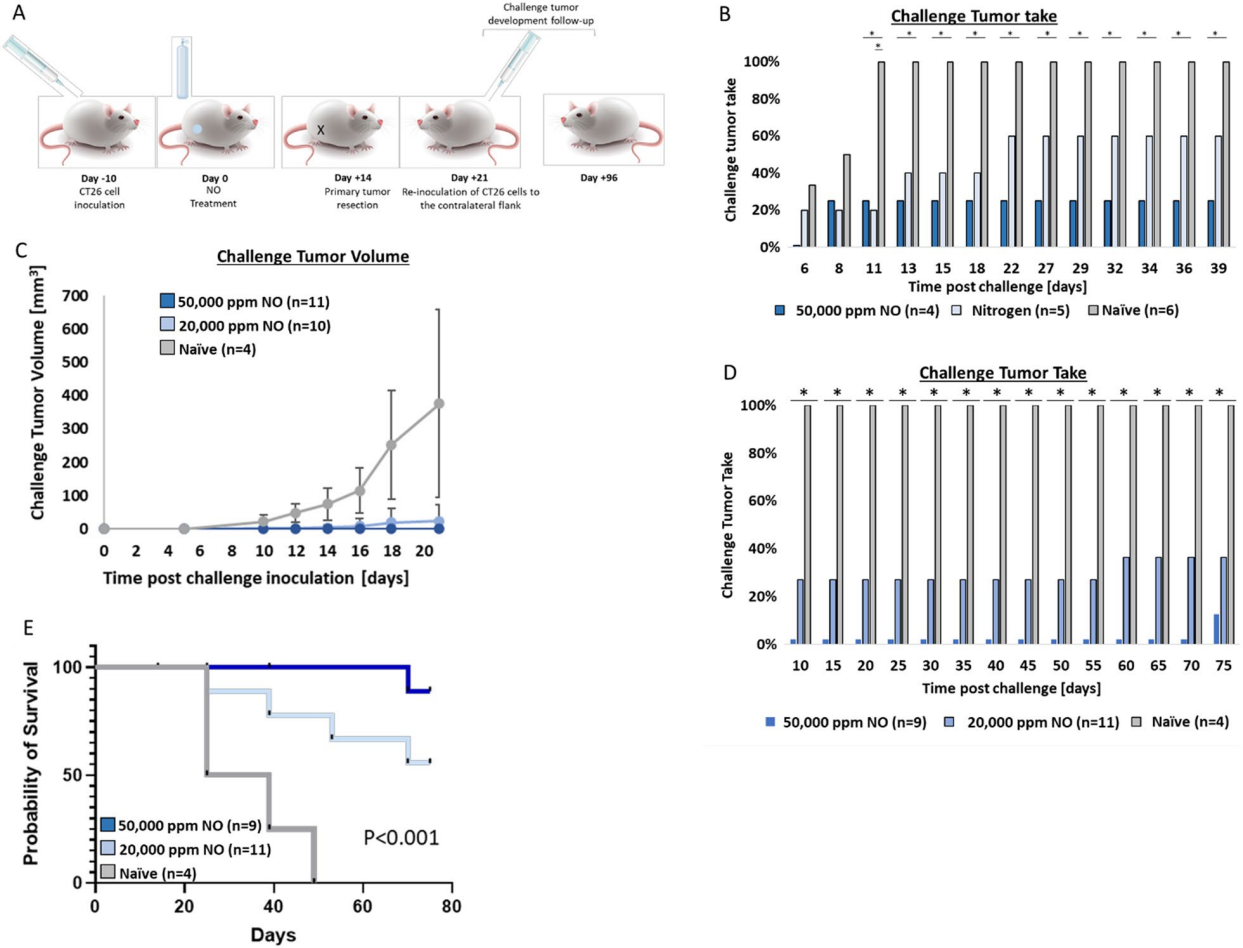
The effect of UHCgNO on CT26 tumor challenge in vivo. **A** The percentage of mice in which the challenge resulted in a new tumor. Chi-square test (naïve *versus*. 50,000 ppm gNO or 20,000 ppm gNO groups) *P* < 0.05 **B** Preliminary challenge assay where mice were treated with 50,000 ppm gNO or nitrogen for 5 min. Two weeks post gas treatment, the tumors were resected and a week later a secondary tumor was induced in the contralateral flank. Fisher’s Exact test, *P < 0.05 **C** Average tumor volume of the challenge tumor. One mouse in the 20,000 ppm arm was excluded from this analysis; this mouse was euthanized on Day 14 post challenge assay start due to tumor volume. **D** Challenge tumor take percentage following either 20,000 ppm gNO, 50,000 ppm gNO, or no treatment. Two mice in the 50,000 ppm arm were excluded from this analysis due to a handling protocol violation following treatment; all other deaths were due to tumor volume triggering euthanasia. The mouse from the 20,000 ppm NO group that was excluded from graph 3C was included in this analysis as the mouse developed a challenge tumor before exclusion from the study **E** Survival graph of gNO -treated and control mice, *P* < 0.001 Mantel Cox logrank test

Twenty-one days after 50,000 ppm gNO treatment of the primary tumor, a secondary CT26 cell inoculation was repeated on the contralateral flank (Fig. 3A). In a preliminary test, CT26 tumor-bearing mice were treated with 50,000 ppm gNO or nitrogen for 5 min. Two weeks post gas treatment, the tumors were resected and a week later a secondary tumor was induced in the contralateral flank. Most (75%) of the mice treated with 50,000 ppm gNO rejected the secondary CT26 cell inoculation, significantly better than the naïve group, while only 40% of the nitrogen-treated mice rejected the secondary tumor, which was not statistically significant (Fig. 3B). Naïve average volume of the challenge tumor in UHCgNO- treated mice was smaller than in the naïve control group (Fig. 3C). Additionally, all gNO-treated mice rejected the secondary cell inoculation, an effect that was maintained for up to 70-days (Fig. 3D). In mice treated with 20,000 ppm gNO, 64% of treated mice rejected a secondary CT26 cell inoculation (Fig. 3D) at 70-days (*P* < 0.05). In contrast, no naïve control mice rejected the challenge tumor, and by day 50 post-treatment (day 29 post-challenge induction), none survived. A significant survival advantage (*P* < 0.001) was achieved in UHCgNO- treated mice compared to the control mice (Fig. 3E).

### Higher T-cell recruitment to UHCgNO-treated tumors compared to nitrogen-treated control

**Fig. 4:**
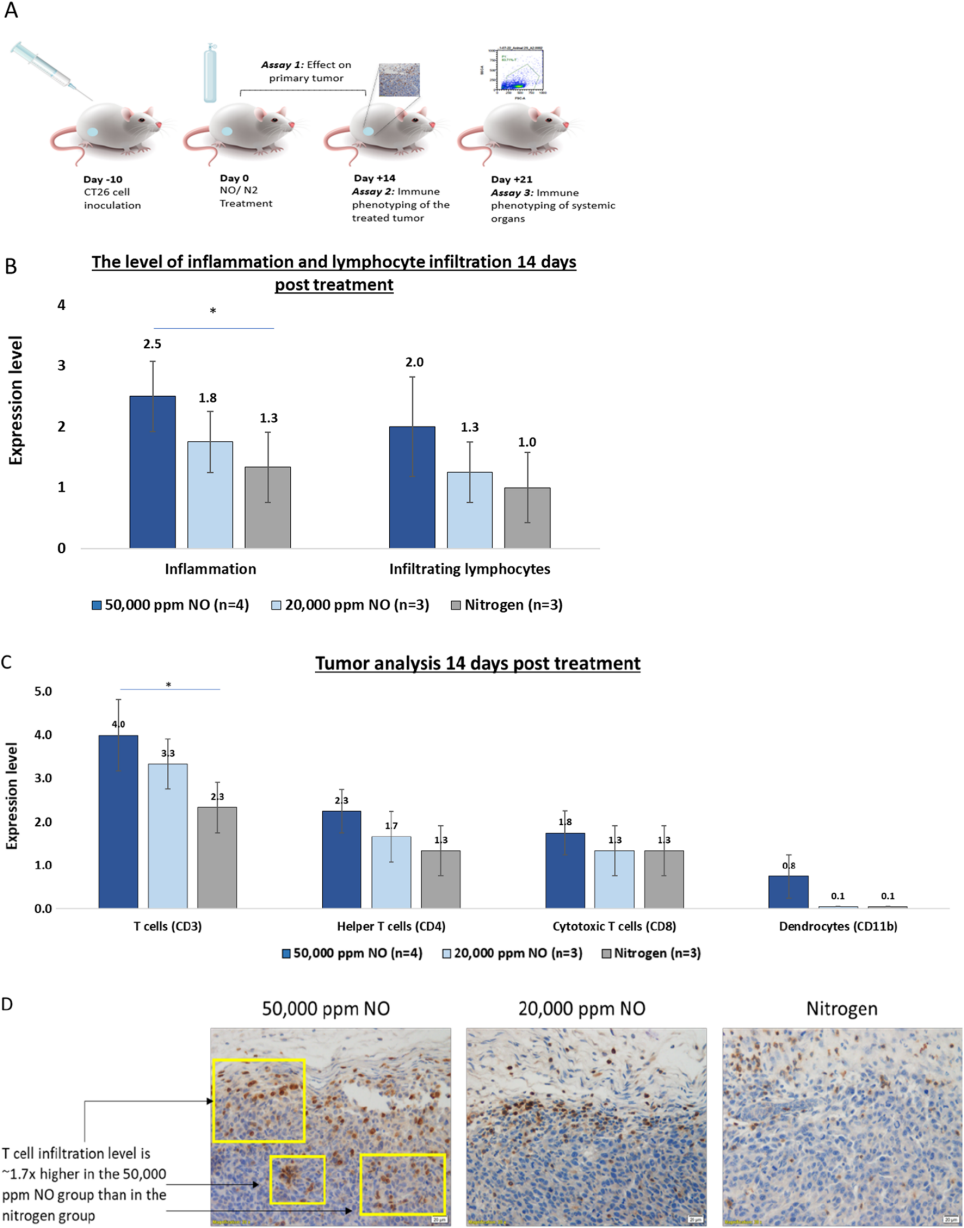
Immune response in tumor following 5 min administration of UHCgNO **A** Assay scheme. **B** The level of inflammation and lymphocytes infiltration 14 days post gas delivery. Inflammation is significantly higher in the 50,000 ppm gNO group versus the nitrogen control (*P < 0.05, one-way ANOVA) **C** Immune cell analysis of treated tumors 14 days post-treatment. The level of CD3 expression is significantly higher in the 50,000 ppm gNO group versus the nitrogen control (*P < 0.05, one-way ANOVA) **D** Representative pictures of tumors stained with anti-CD3. Tumor cells are stained in blue and CD3 + T-cells are stained in brown

The finding that treatment with UHCgNO significantly reduces the chance of tumor formation following injection of cancer cells suggests that UHCgNO induces a durable memory immune response. To examine this hypothesis, adaptive immune cells were assessed 14- and 21-days post-treatment (Fig. 4A). Histological examination on day 14 showed that lymphocytes were recruited to the primary tumor, which correlated to the level of inflammation (Fig. 4B). Analysis of the different immune effectors showed elevated levels of T-cells and dendrocytes in the 50,000 ppm gNO-treated tumors. Levels of T-cell penetration were gNO dose-dependent with 50,000 ppm and 20,000 ppm gNO-treated tumors demonstrating ~ 1.7 × and 1.4 × higher T-cell infiltration levels, respectively, than control-treated tumors (Fig. 4C, D). A similar trend was seen with helper T-cells recruited to the tumor. In the case of cytotoxic T-cells, the highest level of recruitment was observed in the 50,000 ppm gNO-treated group compared to both 20,000 ppm and control-treated tumors (Fig. 4C). Representative slides show that in the UHCgNO-treated groups, T-cells (stained brown) penetrate to the tumor mass, as opposed to the control group in which T-cells are present at the tumor margins (Fig. 4D).

### Elevated systemic immune response following UHCgNO treatment

**Fig. 5:**
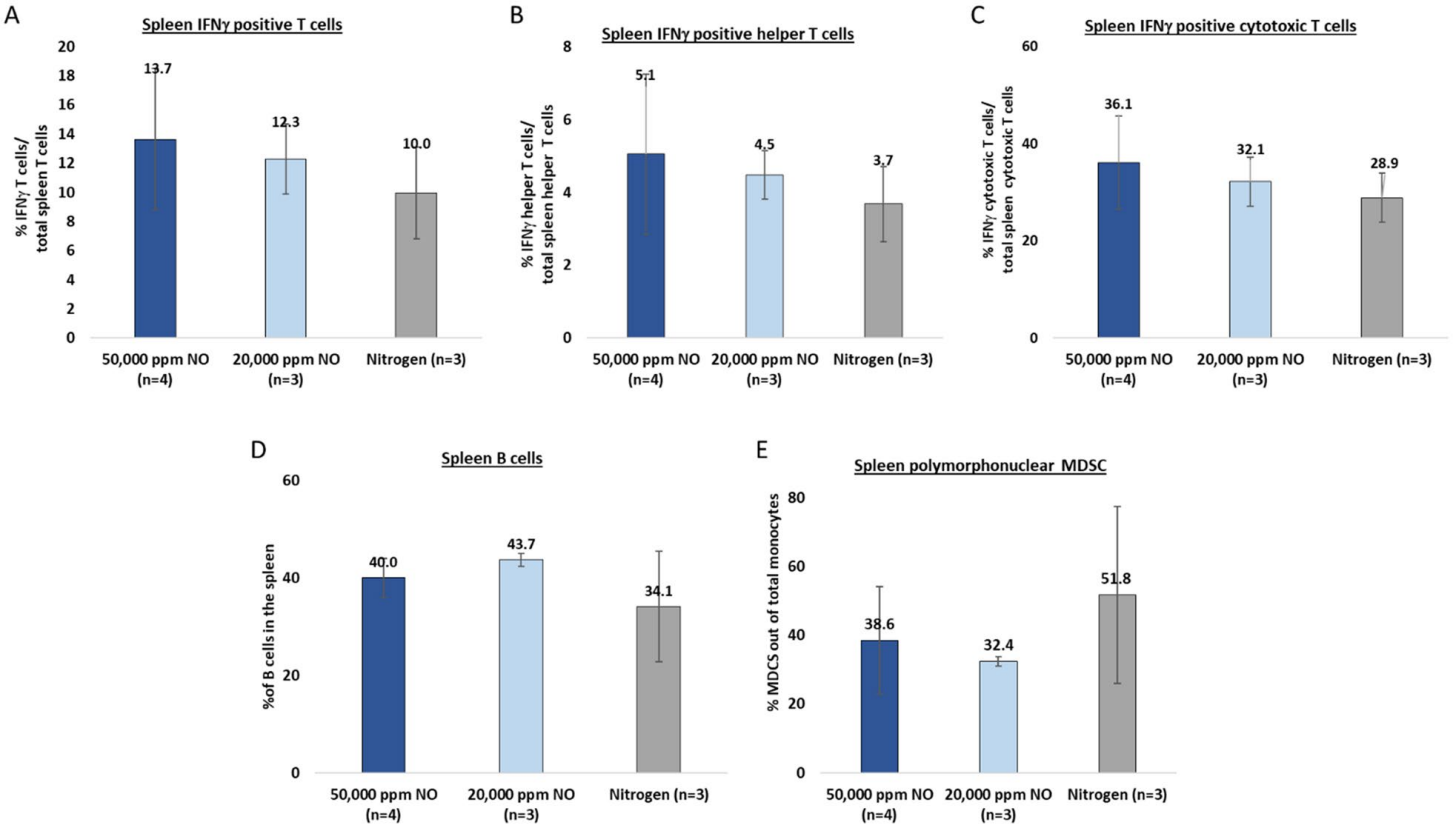
Systemic immune response in the spleen following 5-min exposure to UHCgNO. **A**–**E** The level of immune activation was tested by measuring the amounts of **A** IFNγ positive T-cells out of total T-cells in the spleen. **B** IFNγ positive helper T-cells out of total helper T-cells in the spleen. **C** IFNγ positive cytotoxic T-cells out of total cytotoxic T cells in the spleen. **D** B cells in the spleen. **E** Polymorphonuclear MDSCs out of total monocytes in the spleen

We next investigated whether the local T-cell infiltration was also accompanied by a systemic immune response. For this, T-cells, B-cells, and MDSCs were assessed in the spleen 21-days post-treatment and 7-days after primary tumor resection using flow cytometry (Fig. 4A). To assess the level of T-cell activation, IFNγ positive activated T-cell counts were measured out of total T-cells. Analysis showed that following 50,000 ppm gNO treatment, the ratio of IFNγ-positive T-cells to total T-cells was elevated by 37% compared to the control, while 20,000 ppm gNO increased levels by 23% (Fig. 5A). Similarly, both 50,000 and 20,000 ppm gNO groups showed increased helper T-cell activation by 38% and 22%, respectively (Fig. 5B). Furthermore, cytotoxic T-cells were 25% higher following 50,000 ppm gNO treatment and 11% after 20,000 ppm gNO treatment (Fig. 5C). The levels of B-cells in the spleen were elevated in both treatment groups; however, a dose–response was not observed (Fig. 5D). Immune suppression was also affected by gNO treatment as splenic polymorphonuclear MDSCs were decreased 21 days following 50,000 ppm (25%) or 20,000 ppm gNO treatment (37%) compared to nitrogen control (Fig. 5E).

### Elevated systemic T-cells and B-cells in the blood following UHCgNO treatment

**Fig. 6:**
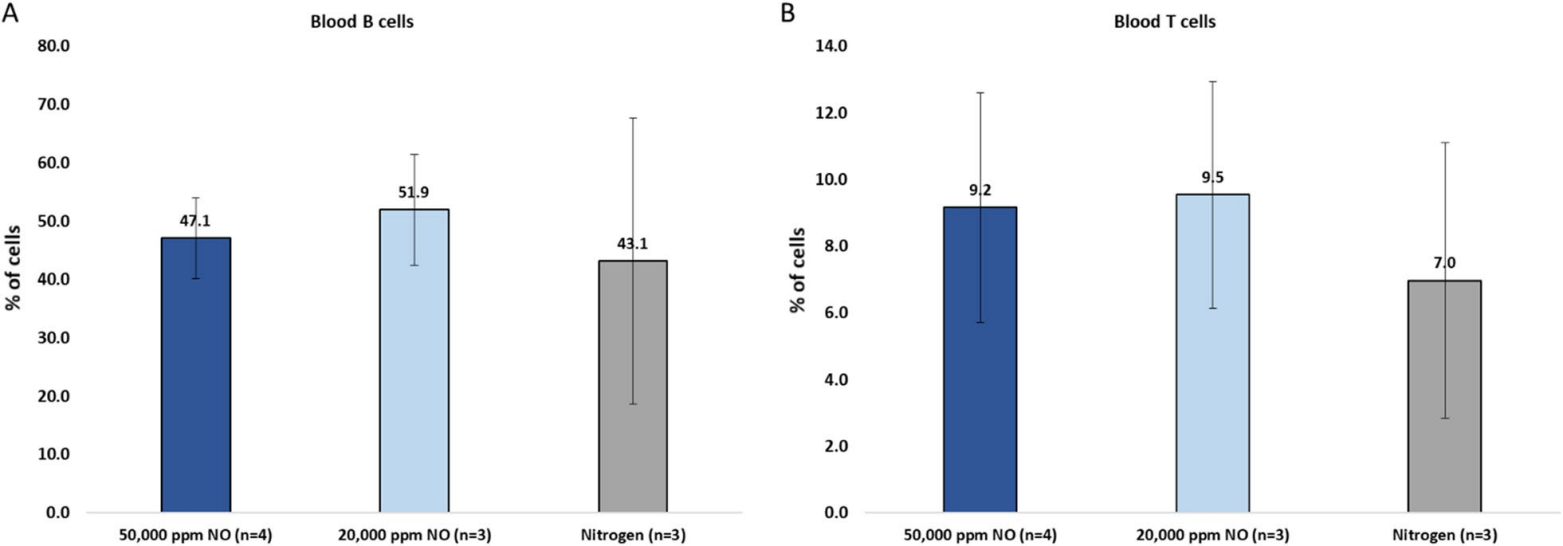
Elevated T-cells and B-cells in the blood following 5-min UHCgNO administration 21 days post-treatment **A** Blood T cell levels. **B** Blood B cell levels

To examine whether the systemic immune response found in the spleen is also apparent in the blood of UHCgNO treated mice, blood T- and B-cell levels were assessed 21-days post-treatment (7-days post-tumor resection). Indeed, blood T-cells levels were elevated in the 50,000 ppm and 20,000 ppm gNO groups by 31% and 36%, respectively (Fig. 6A). In addition, blood B-cell levels increased in the 50,000 ppm and 20,000 ppm gNO groups by 9% and 20%, respectively (Fig. 6B).

## Discussion

Local tumor ablation methods have previously demonstrated an anti-tumor immune response [6–10, 28–33]. For example, Den-Brok et al. showed that 50% of mice rejected a secondary melanoma cell inoculation after treating the primary tumor with cryoablation, and 20% of mice rejected such an inoculation after radiofrequency ablation of the primary tumor [31]. T-cell responses have been observed following microwave ablation in a breast cancer model [32] and following kidney tumor cryoablation in a clinical trial [33]. Other studies have emphasized combining tumor ablation with additional immune-stimulating techniques to increase the ablation-induced immune effects [34–36].

NO has been shown to activate innate and adaptive immune system responses against tumors in a concentration-dependent manner. At high concentrations, NO modulates immune-mediated anti-tumor activities [15]. For example, in lymphoma tumor-bearing mice, NO production in the tumor microenvironment is essential for the anti-tumor activity of CD8 + T-cells [37]. Elevated NO levels in the tumor microenvironment and enhanced inducible NO synthase (iNOS) activity have been associated with elevated anti-tumor activity in a wide array of in vitro models, including pancreatic cancer cell lines, human ovarian cancer cell lines, rodent colon, breast models, and hepatic sarcoma metastases [15]. Furthermore, NO plays a crucial role in the induction of immunogenic cell death in melanoma cells [38].

In the current study, the cytotoxic and immune-stimulating effects of a single, short-term administration of UHCgNO were tested in vitro and in vivo. Three murine cancer models were assessed *in-vitro*: (I) Colon cancer, CT26; (II) Breast cancer, 4T1 and (III) Lewis lung carcinoma, LLC-1. These commonly used cell lines demonstrate diverse tumor-host immune interactions. CT26 has been reported to produce a highly immunogenic tumor microenvironment. Whereas 4T1 has a lower immunogenic nature and is highly metastatic, closely mimicking naturally occurring stage IV human breast cancer [39]. We found that treating these cell types with 25,000–50,000 ppm gNO abolished cell metabolic activity, a marker of viability, proliferation, and cytotoxicity. Although metabolic activity is correlated with cell viability, reduced viability does not always correspond to lethality and may represent cytostatic effects [40]. Cell death analyses, using Annexin-V-PI cell death markers, show that almost all the cells are apoptotic after exposure to 50,000 ppm gNO for 2.5 min. Importantly, the greatest proportion of all cells was found to be in late apoptosis, 97% of CT26 cells, 93% of 4T1, and 99% of LLC-1 cells.

These NO effects on 4T1, CT26 and LLC-1 models align with previously reported results. For example, low NO levels due to decreased endothelial nitric oxide synthase (eNOS) activity, together with decreased phosphorylation, were reported to underlie pulmonary endothelial dysfunction. This pulmonary endothelial dysfunction is an early event in pulmonary metastasis in 4T1 mice models [41]. Interferon-gamma (IFNγ), a known immunomodulatory cytokine, regulates cell proliferation and survival. CT26 cells, which are resistant to IFNγ-mediated cell death, showed re-sensitization to this with the addition of an NO donor [42].

Most importantly, ablating CT26 tumors in vivo via locally administered UHCgNO had long-term immune effects. All mice treated with high NO levels (50,000 ppm gNO) were resistant to developing new tumors when challenged with an additional CT26 cell inoculation. Furthermore, the effect of NO was dose-dependent, with the 20,000 ppm gNO- treated group showing partial resistance up to 70 days following the second inoculation. These results suggest that an anti-tumor immune response induced by UHCgNO treatment prevented secondary CT26 tumor development. Importantly, intratumoral treatment with UHCgNO stimulated an anti-tumor immune response even when the primary tumor was not completely ablated, suggesting that it may be sufficient to destroy only part of the tumor to expose immune cells to cancer antigens. These observations with the CT26 model may be attributed to the highly immunogenic nature of CT26 [43].

Our results suggest that following intratumoral administration of UHCgNO, T-cells, and dendrocytes penetrate the tumor within 14 days. In addition, T and B-cells are elevated in both spleen and blood, and polymorphonuclear MDSCs are reduced in the spleen 21-days post UHCgNO treatment. This upregulation of adaptive immune cells, such as T cells and B cells and downregulation of immune suppressor cells, such as MDSCs may underlie the results of the challenge study.

We hypothesize that ablating the tumor by locally exposing it to high NO levels results in NO-induced apoptosis and necrosis, as observed in other tumor ablation methods. For example, dying tumor cells release DAMPs and recruit antigen-presenting cells (APCs) to process and present tumor antigen to T-cells, leading to their proliferation and maturation and a long-term immune response [44]. The use of UHCgNO as a modality to induce an immune response is an important distinction versus other experimental treatments using NO donors as agents to dilate blood vessels or sensitize tumors to chemotherapies and radiotherapies [21]. We aim to further explore the involvement of specific immune cell populations, such as dendritic cells and macrophages, and the maturation of immune cells, in a time-dependent manner in the future.

Taken together, our findings indicate that local tumor ablation with UHCgNO may induce a potent systemic anti-tumor immune response, mediated by adaptive and innate immune cells, and serve as an innovative therapy with subsequent immune activation against solid tumors.

## Conclusions

In this paper, we presented a new tumor ablation method utilizing UHCgNO followed by the surgical removal of the primary tumor 14 days later, which induces a strong and potent anti-tumor response, apparently via the generation of cancer antigens and their exposure to the patient's immune cells. Our findings indicated that local administration of high NO levels increases tumor infiltration and systemic representation of several adaptive immune cells such as T-cells, dendrocytes and B-cells, together with a decrease in spleen MDSCs. This NO-induced immune response resulted in the significant reduction in the formation of challenge tumors and an improvement in mice survival, implying that the presented UHCgNO ablation method has therapeutic potential as an immunomodulating agent.

## Data Availability

The datasets used and/or analyzed during the current study are available from the corresponding author on reasonable request.

## Abbreviations

ATCC: American type culture collection
CRO: Contract Research Organization
DAMP: Damage associated molecular patterns
IACUC: Institutional Animal Care and Use Committee
IFNγ: Gamma interferon
i.p.: Intraperitoneal
gNO: Gaseous nitric oxide
LPM: Liters per minute
MDSCs: Myeloid-derived suppressor cells
MSI: Microsatellite instability
NOS: Nitric oxide synthase
ON: Overnight
Ppm: Parts per million
S.C.UHCgNO: Subcutaneous ultra-high concentration gaseous nitric oxide

## References

[1] Anderson RL, Balasas T, Callaghan J, Coombes RC, Evans J, Hall JA (2019). A framework for the development of effective anti-metastatic agents. Nat Rev Clin Oncol.

[2] Steeg PS (2016). Targeting metastasis. Nat Rev Cancer.

[3] Qian C-N, Mei Y, Zhang J (2017). Cancer metastasis: issues and challenges. Chin J Cancer.

[4] Pardoll DM (2012). The blockade of immune checkpoints in cancer immunotherapy. Nat Rev Cancer.

[5] Horvath L, Thienpont B, Zhao L, Wolf D, Pircher A (2020). Overcoming immunotherapy resistance in non-small cell lung cancer (NSCLC)—novel approaches and future outlook. Mol Cancer.

[6] Keisari Y, Hochman I, Confino H, Korenstein R, Kelson I (2014). Activation of local and systemic anti-tumor immune responses by ablation of solid tumors with intratumoral electrochemical or alpha radiation treatments. Cancer Immunol Immunother.

[7] Confino H, Hochman I, Efrati M, Schmidt M, Umansky V, Kelson I (2015). Tumor ablation by intratumoral Ra-224-loaded wires induces anti-tumor immunity against experimental metastatic tumors. Cancer Immunol Immunother.

[8] Slovak R, Ludwig JM, Gettinger SN, Herbst RS, Kim HS (2017). Immuno-thermal ablations–boosting the anticancer immune response. J Immunother Cancer.

[9] van den Bijgaart RJ, Eikelenboom DC, Hoogenboom M, Fütterer JJ, den Brok MH, Adema GJ (2017). Thermal and mechanical high-intensity focused ultrasound: perspectives on tumor ablation, immune effects and combination strategies. Cancer Immunol Immunother.

[10] OBrien MA, Power DG, Clover AJP, Bird B, Soden DM, Forde PF (2014). Local tumour ablative therapies: opportunities for maximising immune engagement and activation. Biochimica et Biophysica Acta.

[11] Knavel EM, Brace CL (2013). Tumor ablation: common modalities and general practices. Tech Vasc Interv Radiol.

[12] Zhu J, Xu Y, Lu XJ (2019). Stereotactic body radiation therapy and ablative therapies for solid tumors: recent advances and clinical applications. Technol Cancer Res Treat.

[13] Kang TW, Rhim H (2015). Recent advances in tumor ablation for hepatocellular carcinoma. Liver Cancer.

[14] Huerta S (2015). Nitric oxide for cancer therapy. Future Sci OA.

[15] Vannini F, Kashfi K, Nath N (2015). The dual role of iNOS in cancer. Redox Biol.

[16] Seabra AB, Durán N (2018). Nitric oxide donors for prostate and bladder cancers: current state and challenges. Eur J Pharmacol.

[17] Alimoradi H, Greish K, Gamble AB, Giles GI (2019). Controlled delivery of nitric oxide for cancer therapy. Pharm Nanotechnol.

[18] Ning S, Bednarski M, Oronsky B, Scicinski J, Knox SJ (2014). Novel nitric oxide generating compound glycidyl nitrate enhances the therapeutic efficacy of chemotherapy and radiotherapy. Biochem Biophys Res Commun.

[19] Bonavida B, Baritaki S, Huerta-Yepez S, Vega MI, Chatterjee D, Yeung K (2008). Novel therapeutic applications of nitric oxide donors in cancer: roles in chemo-and immunosensitization to apoptosis and inhibition of metastases. Nitric Oxide.

[20] Burke AJ, Sullivan FJ, Giles FJ, Glynn SA (2013). The yin and yang of nitric oxide in cancer progression. Carcinogenesis.

[21] Mintz J, Vedenko A, Rosete O, Shah K, Goldstein G, Hare JM, Ramasamy R, Arora H (2021). Current advances of nitric oxide in cancer and anticancer therapeutics. Vaccines.

[22] Thomas DD (2015). Breathing new life into nitric oxide signaling: a brief overview of the interplay between oxygen and nitric oxide. Redox Biol.

[23] Therapeutics I. INOMAX nitric-oxide-gas. Food and Drug Administration (FDA). 2013.

[24] European public assessment report (EPAR) for INOmax. European Medicines Agency (EMA) (2021). Accessed 2021.

[25] Schairer DO, Chouake JS, Nosanchuk JD, Friedman AJ (2012). The potential of nitric oxide releasing therapies as antimicrobial agents. Virulence.

[26] Stasko N, McHale K, Hollenbach SJ, Martin M, Doxey R (2018). Nitric oxide-releasing macromolecule exhibits broad-spectrum antifungal activity and utility as a topical treatment for superficial fungal infections. Antimicrob Agents Chemother.

[27] Tandon M, Wu W, Moore K, Winchester S, Tu YP, Miller C (2022). SARS-CoV-2 accelerated clearance using a novel nitric oxide nasal spray (NONS) treatment: a randomized trial. Lancet Reg Health Southeast Asia.

[28] Thakur A, Littrup P, Paul EN, Adam B, Heilbrun LK, Lum LG (2011). Induction of specific cellular and humoral responses against renal cell carcinoma after combination therapy with cryoablation and granulocyte-macrophage colony stimulating factor: a pilot study. J Immunother.

[29] McArthur HL, Diab A, Page DB, Yuan J, Solomon SB, Sacchini V (2016). A pilot study of preoperative single-dose ipilimumab and/or cryoablation in women with early-stage breast cancer with comprehensive immune profiling. Clin Cancer Res.

[30] Shi L, Chen L, Wu C, Zhu Y, Xu B, Zheng X (2016). PD-1 blockade boosts radiofrequency ablation–elicited adaptive immune responses against tumor. Clin Cancer Res.

[31] den Brok MH, Sutmuller RP, van der Voort R, Bennink EJ, Figdor CG, Ruers TJ (2004). In situ tumor ablation creates an antigen source for the generation of antitumor immunity. Can Res.

[32] Li L, Wang W, Pan H, Ma G, Shi X, Xie H (2017). Microwave ablation combined with OK-432 induces Th1-type response and specific antitumor immunity in a murine model of breast cancer. J Transl Med.

[33] Kato T, Iwasaki T, Uemura M, Nagahara A, Higashihara H, Osuga K (2017). Characterization of the cryoablation-induced immune response in kidney cancer patients. Oncoimmunology.

[34] Levy MY, Sidana A, Chowdhury WH, Solomon SB, Drake CG, Rodriguez R (2009). Cyclophosphamide unmasks an antimetastatic effect of local tumor cryoablation. J Pharmacol Exp Ther.

[35] Yamada T, Tateishi R, Iwai M, Koike K, Todo T (2020). Neoadjuvant use of oncolytic herpes virus G47Δ enhances the antitumor efficacy of radiofrequency ablation. Mol Ther Oncolytics.

[36] Eranki A, Srinivasan P, Ries M, Kim A, Lazarski CA, Rossi CT (2020). High-intensity focused ultrasound (HIFU) triggers immune sensitization of refractory murine neuroblastoma to checkpoint inhibitor therapy. Clin Cancer Res.

[37] Marigo I, Zilio S, Desantis G, Mlecnik B, Agnellini AH, Ugel S (2016). T cell cancer therapy requires CD40-CD40L activation of tumor necrosis factor and inducible nitric-oxide-synthase-producing dendritic cells. Cancer Cell.

[38] Lin A, Gorbanev Y, De Backer J, Van Loenhout J, Van Boxem W, Lemière F (2019). Non-thermal plasma as a unique delivery system of short-lived reactive oxygen and nitrogen species for immunogenic cell death in melanoma cells. Adv Sci.

[39] Chen L, Huang T-G, Meseck M, Mandeli J, Fallon J, Woo SL (2007). Rejection of metastatic 4T1 breast cancer by attenuation of Treg cells in combination with immune stimulation. Mol Ther.

[40] Gupta A, Gautam P, Wennerberg K, Aittokallio T (2020). A normalized drug response metric improves accuracy and consistency of anticancer drug sensitivity quantification in cell-based screening. Commun Biol.

[41] Smeda M, Kieronska A, Adamski MG, Proniewski B, Sternak M, Mohaissen T (2018). Nitric oxide deficiency and endothelial–mesenchymal transition of pulmonary endothelium in the progression of 4T1 metastatic breast cancer in mice. Breast Cancer Res.

[42] Rakshit S, Chandrasekar BS, Saha B, Victor ES, Majumdar S, Nandi D (2014). Interferon-gamma induced cell death: regulation and contributions of nitric oxide, cJun N-terminal kinase, reactive oxygen species and peroxynitrite. Biochimica et Biophysica Acta.

[43] Lechner MG, Karimi SS, Barry-Holson K, Angell TE, Murphy KA, Church CH (2013). Immunogenicity of murine solid tumor models as a defining feature of in vivo behavior and response to immunotherapy. J Immunother.

[44] Jiang H-R, Gilham DE, Mulryan K, Kirillova N, Hawkins RE, Stern PL (2006). Combination of vaccination and chimeric receptor expressing T cells provides improved active therapy of tumors. J Immunol.

